# Late Onset Bipolar Disorder (LOBD): a case report

**DOI:** 10.1192/j.eurpsy.2024.1317

**Published:** 2024-08-27

**Authors:** M. Martín De Argila Lorente, B. Franco Lovaco, B. Rabinovici, C. Díaz Mayoral, G. E. Toapanta Yanza

**Affiliations:** ^1^Psychiatry, Hospital Dr. Rodríguez Lafora; ^2^Psychiatry, Hospital Universitario Príncipe de Asturias; ^3^Geriatric, Hospital Universitario La Paz, Madrid, Spain

## Abstract

**Introduction:**

Bipolar disorder (BD) in the elderly patient may present as the evolution of illness initiated earlier in life or as a new-onset entity. Therefore, two groups of patients are distinguished: “late onset” (LOBD) when the first mania occurs in old age and “early onset” in elderly patients with long-standing history. BD in elderly patients (**≥**60 years) constitutes 25% of all BD cases. Specific aspects of older age bipolar disorder (OABD) are somatic and psychiatric comorbidity, impaired cognition and age-related psychosocial functioning. The management of BD in the elderly is complex given the high sensitivity of these patients to pharmacological side effects, particularly of psychotropic drugs.

**Objectives:**

The case of a patient with LOBD is presented, followed by a theoretical review of the subject.

**Methods:**

A case is presented with a bibliographic review.

**Results:**

A 76-year-old woman who had no prior history of mental health issues until March 2023 when she was initially admitted to a geriatric hospitalization unit for manifesting manic symptoms. She was readmitted in July 2023 due to worsening depressive symptoms that included a declining mood, passive thoughts of death, deterioration in self-care, weight loss, insomnia, constipation, and dry mouth despite recent changes in her medications. She was on treatment with escitalopram (which was gradually discontinued and replaced with mirtazapine), quetiapine, lormetazepam, and lorazepam. Imaging tests showed chronic ischemic lesions in her brain and a small meningioma, the rest of the test were normal.

The initial diagnostic hypothesis was a bipolar depressive episode, and her treatment was adjusted accordingly. She was started on lithium, and her quetiapine dosage was increased, along with the anxiolytic lorazepam. Due to the persistence of depressive symptoms, including low mood, anhedonia, apathy, and negative thoughts, she was also prescribed antidepressant medication (venlafaxine and mirtazapine). Her condition gradually improved, with better eating and sleep patterns, increased participation in activities, and reduced somatic complaints and anxiety.

As she continued to experience somnolence and decreased morning energy, her antipsychotic medication was switched from quetiapine to lurasidone. The dose of lithium was decreased due to tremors in her extremities, although they remained within the therapeutic range. Despite these adjustments, her mood significantly improved, and she showed no signs of worsening or psychotic symptoms, leading to her discharge.

**Conclusions:**

Summarizing different studies, LOBD who develop mania for the first time at an advanced age (**≥** 50 years) constitute 5-10% of all BD. It is important to perform a thorough differential diagnosis, as an organic substrate and diverse etiologies may be present. Current guidelines recommend that first-line treatment of OABD should be similar to that of BD in young patients, with careful use of psychotropic drugs.

**Disclosure of Interest:**

None Declared

